# Supplementary material for: Persistent inflammation during anti-tuberculosis treatment with diabetes comorbidity
Source: eLife. 2019 Jul 4;8:e46477. doi: 10.7554/eLife.46477 (PMC6660216; doi:10.7554/eLife.46477)
Supplement: Transparent reporting form [file elife-46477-transrepform.docx]

***eLife’s* transparent reporting form**

We encourage authors to provide detailed information *within their submission* to facilitate the interpretation and replication of experiments. Authors can upload supporting documentation to indicate the use of appropriate reporting guidelines for health-related research (see [EQUATOR Network](http://www.equator-network.org/%20)), life science research (see the [BioSharing Information Resource](https://biosharing.org/)), or the [ARRIVE guidelines](http://www.plosbiology.org/article/info:doi/10.1371/journal.pbio.1000412) for reporting work involving animal research. Where applicable, authors should refer to any relevant reporting standards documents in this form.

If you have any questions, please consult our Journal Policies and/or contact us: [editorial@elifesciences.org](mailto:editorial@elifesciences.org).

**Sample-size estimation**

- You should state whether an appropriate sample size was computed when the study was being designed
- You should state the statistical method of sample size computation and any required assumptions
- If no explicit power analysis was used, you should describe how you decided what sample (replicate) size (number) to use

Please outline where this information can be found within the submission (e.g., sections or figure legends), or explain why this information doesn’t apply to your submission:

The effect sizes noted in our preliminary data provide strong guidance regarding power and sample size for the comparisons of cytokine responses (Andrade et al. Chest 2014) Sample size and power are therefore based on this endpoint. With 20 cases (TB with diabetes) and 20 controls (TB without diabetes), we will have approximately 80 % power to detect a 2.5-fold difference in levels of at least 1 cytokine, with a significance level α of 0.05. Such an effect size is reasonable given our preliminary data. We decided to run the Indian data as training/discovery set and increased the sample size to increase power to detect smaller fold differences (1.5), keeping the study power at 80% and α of 0.05. The Brazilian data was treated as validation set and we kept sample size close to what the initial study power calculation indicated.

**Replicates**

- You should report how often each experiment was performed
- You should include a definition of biological versus technical replication
- The data obtained should be provided and sufficient information should be provided to indicate the number of independent biological and/or technical replicates
- If you encountered any outliers, you should describe how these were handled
- Criteria for exclusion/inclusion of data should be clearly stated
- High-throughput sequence data should be uploaded before submission, with a private link for reviewers provided (these are available from both GEO and ArrayExpress)

Please outline where this information can be found within the submission (e.g., sections or figure legends), or explain why this information doesn’t apply to your submission:

Each plasma sample for Luminex was assayed in duplicate.

**Statistical reporting**

- Statistical analysis methods should be described and justified
- Raw data should be presented in figures whenever informative to do so (typically when N per group is less than 10)
- For each experiment, you should identify the statistical tests used, exact values of N, definitions of center, methods of multiple test correction, and dispersion and precision measures (e.g., mean, median, SD, SEM, confidence intervals; and, for the major substantive results, a measure of effect size (e.g., Pearson's r, Cohen's d)
- Report exact p-values wherever possible alongside the summary statistics and 95% confidence intervals. These should be reported for all key questions and not only when the p-value is less than 0.05.

Please outline where this information can be found within the submission (e.g., sections or figure legends), or explain why this information doesn’t apply to your submission:

Please referrer to the Materials and Methods subsection “Statistical Analysis” found on page 15 of the submitted full manuscript.

(For large datasets, or papers with a very large number of statistical tests, you may upload a single table file with tests, Ns, etc., with reference to sections in the manuscript.)

**Group allocation**

- Indicate how samples were allocated into experimental groups (in the case of clinical studies, please specify allocation to treatment method); if randomization was used, please also state if restricted randomization was applied
- Indicate if masking was used during group allocation, data collection and/or data analysis

Please outline where this information can be found within the submission (e.g., sections or figure legends), or explain why this information doesn’t apply to your submission:

Individuals newly diagnosed with pulmonary TB were recruited sequentially at the study sites in India and Brazil. Allocation into groups with and without concurrent diabetes mellitus was based on oral glucose tolerance test or HbA1c, as described in Material and Methods, subsections “Indian Stud Population” and “Brazilian Study Population” on pages 13-14 of the submitted full manuscript.

**Additional data files (“source data”)**

- We encourage you to upload relevant additional data files, such as numerical data that are represented as a graph in a figure, or as a summary table
- Where provided, these should be in the most useful format, and they can be uploaded as “Source data” files linked to a main figure or table
- Include model definition files including the full list of parameters used
- Include code used for data analysis (e.g., R, MatLab)
- Avoid stating that data files are “available upon request”

Please indicate the figures or tables for which source data files have been provided:

Source data for Figures 1 through 5 are included in the two uploaded .xlcx files “Brazil Data” and “India Data”.
